# QSIM: quantitative structured illumination microscopy image processing in ImageJ

**DOI:** 10.1186/1475-925X-14-4

**Published:** 2015-01-14

**Authors:** Liang Gao

**Affiliations:** Department of Biomedical Engineering, Washington University, St. Louis, MO 63139 USA

**Keywords:** Structured illumination microscopy, Quantitative imaging, 3D imaging, ImageJ

## Abstract

**Background:**

Structured illumination microscopy has been extensively used in biological imaging due to its low cost and easy implementation. However, the lack of quantitative imaging capability limits its application in absolute irradiance measurements.

**Method:**

We have developed a quantitative structured illumination microscopy image processing algorithm (QSIM) as a plugin for the widely used ImageJ software. QSIM can work with the raw images acquired by a traditional structured illumination microscope and can quantitatively measure photon numbers, with noise estimates for both wide-field images and sectioned images.

**Results and conclusion:**

We demonstrated the quantitative image processing capability of QSIM by imaging a mouse kidney section in 3D. The results show that QSIM can transform structured illumination microscopy from qualitative to quantitative, which is essential for demanding fluorescence imaging applications.

**Electronic supplementary material:**

The online version of this article (doi:10.1186/1475-925X-14-4) contains supplementary material, which is available to authorized users.

## Background

Structured illumination microscopy (SIM), a three-dimensional (3D) optical imaging technique, has been widely used in biomedical research because of its relatively low cost and easy implementation [1–4]. Currently, SIM is commercially available as an add-on module (e.g., Zeiss Apotome) for most wide-field optical microscopes, enabling acquisition of high resolution sectioned images, much as a scanning confocal microscope does [5, 6].

To acquire a sectioned depth layer, SIM normally captures three images *I*_1_, *I*_2_, *I*_3_ with 2*π*/3 phase shifted sinusoid illumination patterns, and computes the sectioned image as [1]
1

However, this demodulation algorithm has been criticized as non-quantitative because it lacked a means of converting the raw detected signal into photon counts. For a shot-noise-limited imaging system, e.g., a confocal microscope, imaging a uniform illuminated field, the detected photons *N*_*d*_ simply equal
2

where  is the mean of the intensity grey level distribution and σ is its standard deviation [7]. Equation 2 is valid because in a shot-noise-limited imaging system the image noise σ obeys a Poisson distribution. For SIM, however, the calculation of *N*_*d*_ cannot follow the same approach because the out-of-focus light is removed after being detected. In fact, the image noise σ in SIM is attributed to three sources – photon noise from the sectioned depth layer, photon noise from the out-of-focus depth layers, and noise caused by nonlinear demodulation (Eq. 1).

The lack of quantification limits SIM’s application in absolute irradiance measurements [8, 9]. To overcome this problem, we recently proposed a quantitative SIM image reconstruction algorithm [10]. By calibrating the camera’s gain and estimating the modulation contrast in the detected images, the number of detected photons from a single sectioned depth layer can be derived. This gives SIM the same quantitative capability to measure photons as a confocal microscope. To make our algorithm easily available to the biological research community, here we present QSIM, a free open-source plugin for the widely used ImageJ software (http://rsb.info.nih.gov/ij/). QSIM integrates system calibration and image processing in one software package. By performing two simple calibration experiments prior to SIM imaging (Implementation), QSIM is able to calculate photons and noise maps for both reconstructed sectioned and wide-field images.

## Implementation

**Figure 1:**
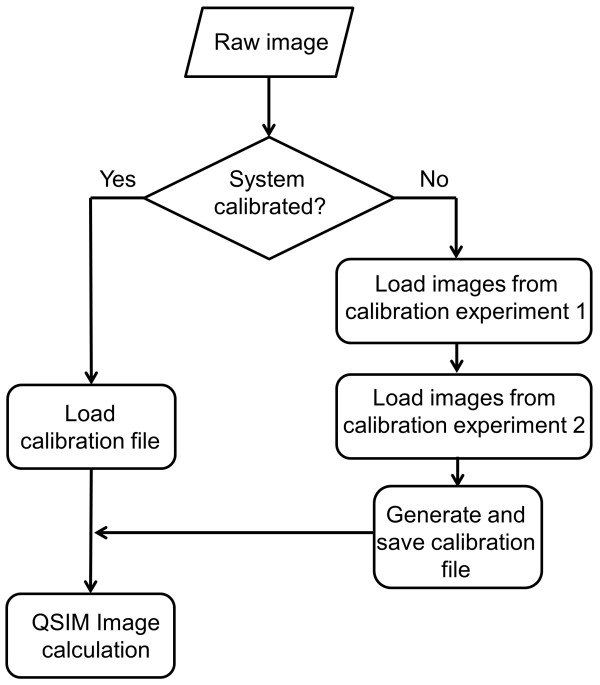
**QSIM image processing flowchart.**

The image processing flowchart of QSIM is shown in Figure 1. To employ QSIM in quantitative SIM image processing, two calibration experiments are required, involving camera gain measurement and image contrast modulation measurement. The underpinning principle of QSIM calibration is detailed in [10]. Here we focus on its practical implementation on a microscopic imaging system and procedures to acquire the calibration data for the software.

### Calibration experiment 1: camera gain measurement

Step 1: Set up Koehler illumination on the microscope sample stage.

Step 2: Choose the camera gain settings and digitization level that will be used in SIM imaging experiments.

Step 3: Image a uniformly illuminated field with a microscope objective. Increase the illumination intensity step by step from zero to the level where the camera is almost saturated. The number of illumination steps should be larger than five. At each illumination level, capture two empty field images with the same integration time.

### Calibration experiment 2: illumination modulation contrast measurement

Step 1: Prepare an evenly-distributed fluorescent microsphere sample. Choose fluorescence microspheres with mean diameters less than the targeted sectioning thickness. Uniformly suspend the fluorescence microspheres by vortex mixing and sonicating the suspension. Deposit the suspension onto a microscope slide and seal it by a coverslip.

Step 2: Image the microsphere slide with SIM, and save the raw images (unprocessed with grid patterns). A separate measurement is required for each objective and grid combination because the modulation contrast is specific to the microscope’s objective and illumination grid’s frequency.

**Figure 2:**
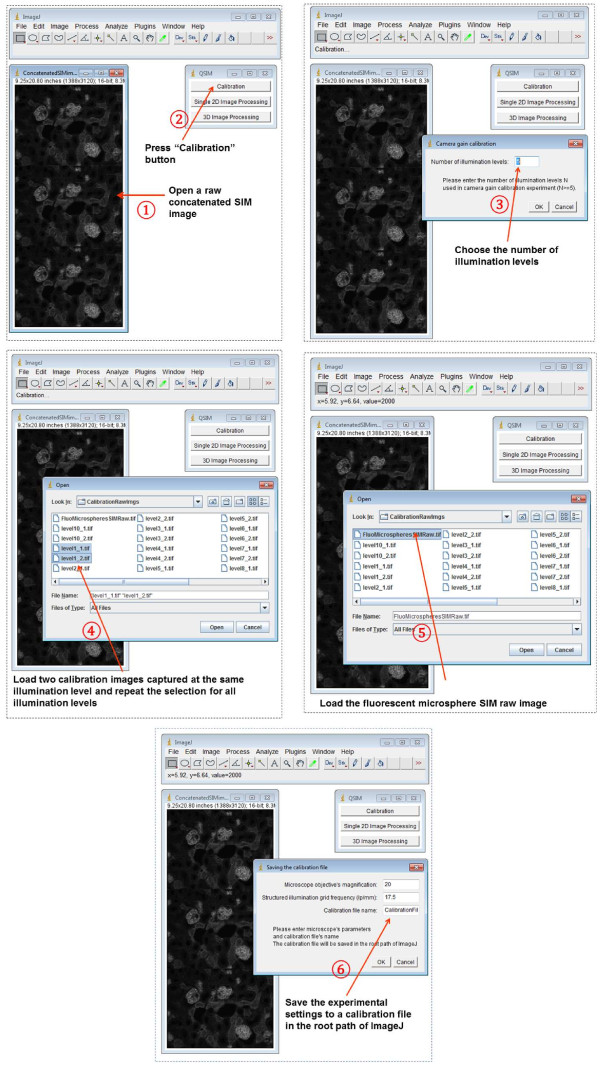
**Screenshots of operating QSIM’s “Calibration” module.**

**Figure 3:**
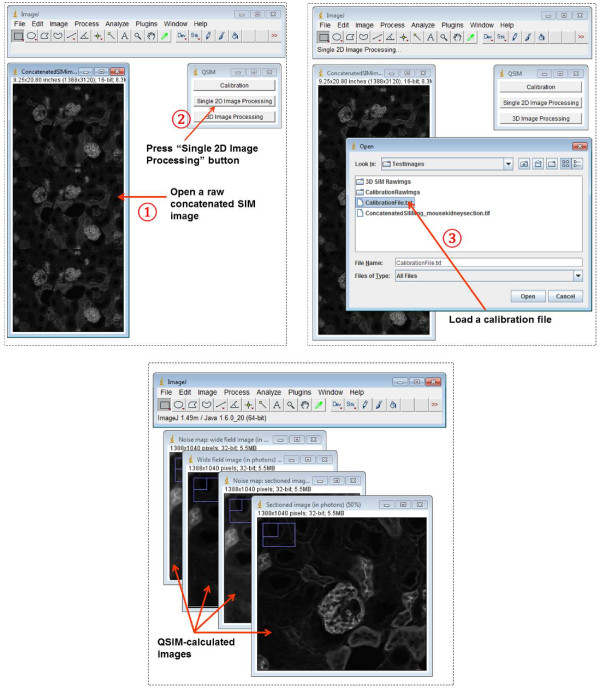
**Screenshots of operating QSIM’s “Single 2D Image Processing” module.**

**Figure 4:**
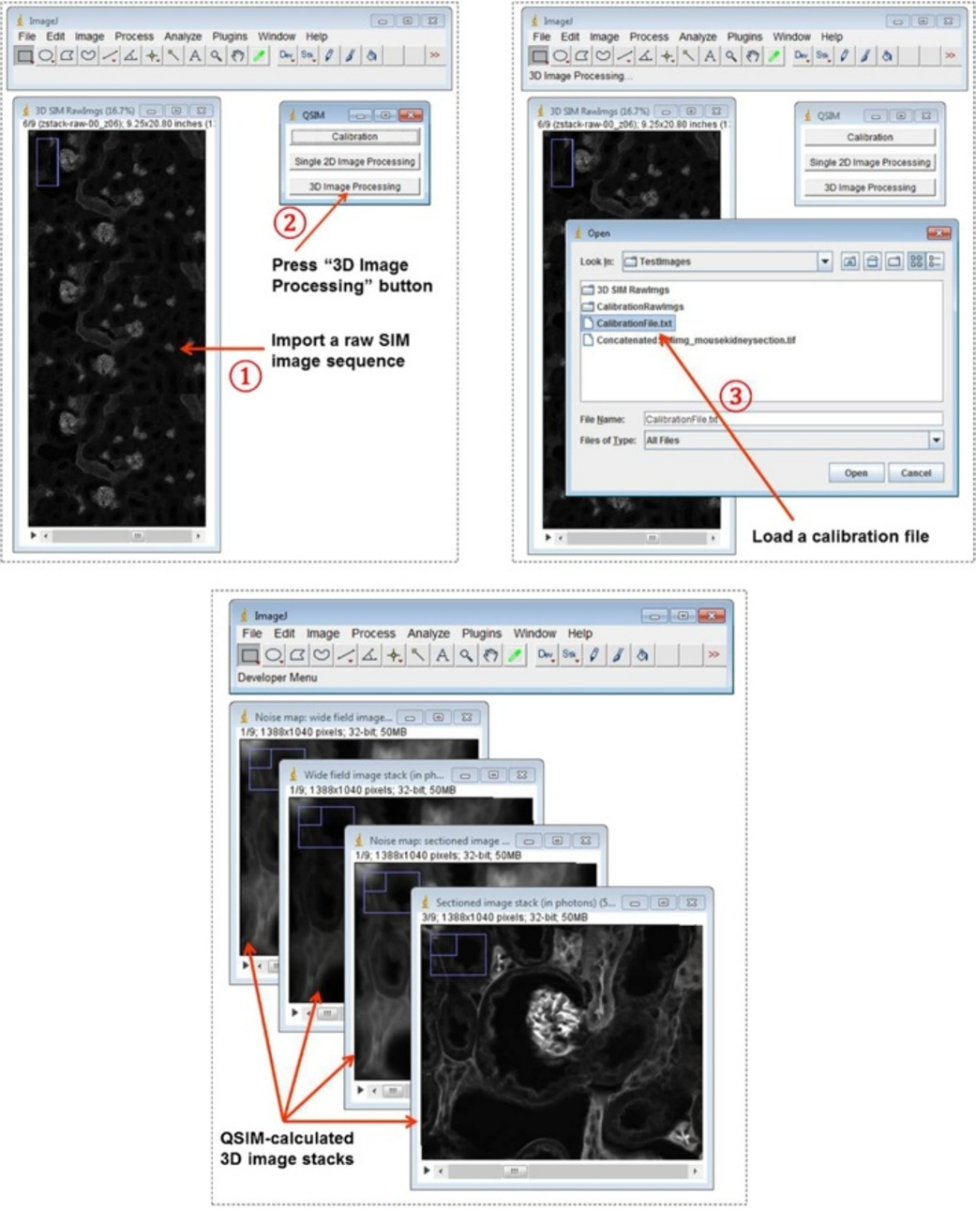
**Screenshots of operating QSIM’s “3D Image Processing” module.**

After these two sets of images (from experiment 1 and 2) are loaded, QSIM automatically calculates the camera gain *g* and illumination modulation contrast *m*, and saves the results into a calibration file. This file can be used for processing SIM images acquired with the same settings as the calibration experiment. QSIM has three modules: calibration, single 2D image processing, and 3D image processing. The screenshots of operating these modules are shown in Figures 2, 3 and 4, respectively. Provided that each SIM phase image has *M* × *N* (rows × columns) pixels, for 2D image processing, QSIM requires a single image—in which three SIM phase images are concatenated into an array of 3*M* × *N* pixels—as the data entry. For 3D image processing, QSIM requires a sequence of similarly constructed SIM images, and each concatenated image must be captured at a different depth. QSIM calculates four images—wide-field and sectioned fluorescent images and their corresponding noise maps—as the data output at each depth layer. All these images have *M* × *N* pixels, and the pixel values are in the unit of photons.

## Results and discussion

To demonstrate the advantages of QSIM in processing biological SIM images, we imaged a mouse kidney section (~10 μm thick, Life technologies) on a Zeiss Axio Imager Z1 microscope equipped with an Apotome module and an AxioCam MRM monochromatic camera (1388 × 1040 pixels). The tissue sample was stained with a fluorescent dye, Alexa Fluor 488 (495 nm excitation maximum, 519 nm emission maximum), on convoluted tubules and illuminated by a HBO100 light source. The fluorescence was collected by a Zeiss Plan-Apochromatic 20× objective with a numerical aperture of 0.8. The acquisition of raw SIM images was accomplished by Zeiss Apotome’s companion operating software, AxioVision.

**Figure 5:**
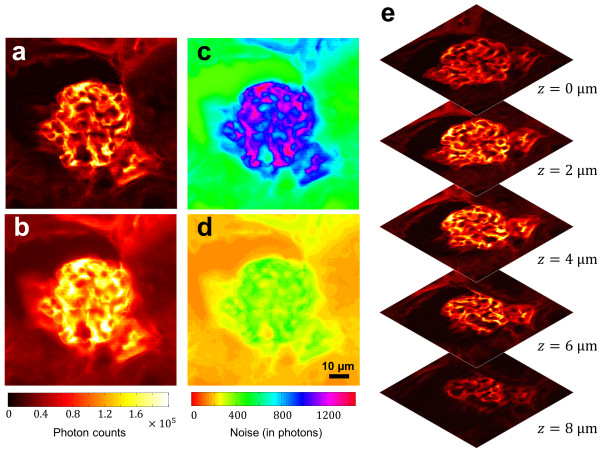
**QSIM-calculated quantitative images. (a)** Sectioned mouse kidney image at z = 4 μm. **(b)** Wide-field image at z = 4 μm. **(c)** Corresponding noise map of sectioned image. **(d)** Corresponding noise map of wide-field image. **(e)** Sectioned images at all depth layers. The image intensity values are in units of photons.

The acquired raw SIM images were then loaded into ImageJ and processed by the QSIM module. The QSIM-calculated wide-field image, sectioned image, and their noise maps at a representative depth *z* = 4 *μm* are shown in Figure 5a-d, respectively. In addition, we scanned the sample along the depth axis with a step size of 2 μm, and show the corresponding sectioned images in Figure 5e. It is important to note that in the QSIM-calculated images the pixels’ values are in units of photons, rather than in an arbitrary grey level unit as commonly seen in existing SIM image processing software, such as AxioVision. This capability of measuring absolute irradiance is crucial for leveraging SIM in quantitative fluorescence imaging applications, such as fluorescence resonance energy transfer [11], fluorescence correlation spectroscopy [12], and quantum yield measurement experiments [13]. Moreover, QSIM calculated the noise at each pixel for both the wide-field and sectioned images, again in units of photons. The resultant noise maps (Figure 5c and d) thereby provide users with a tool to assess the value of measured results.

**Figure 6:**
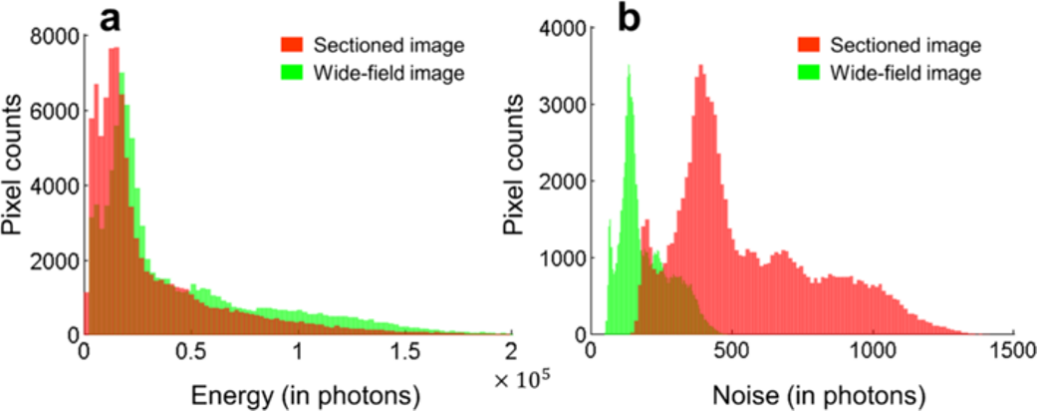
**Histograms of QSIM-calculated sectioned image and wide-field image. (a)** Histogram of light energy. **(b)** Histogram of noise.

To further evaluate the images calculated by QSIM, we calculated the histograms of the measured photons and noise map for the sectioned image (Figure 6a) and wide-field image (Figure 6b). The results indicate that, compared to the wide-field image, the sectioned image has more “dark” pixels because SIM removes the background and thereby increases the image contrast. In addition, the sectioned image also has more “noisy” pixels, degrading the system’s performance from the shot-noise limit. This is consistent with the fact that, although the out-of-focus light is subtracted from the sectioned image, the added shot noise contributed by the out-of-focus light cannot be eliminated. Instead, this noise is amplified by a factor of  (*m* is the illumination modulation contrast at the sample plane) due to SIM’s nonlinear demodulation [10].

## Conclusions

In summary, using QSIM software in conjunction with a traditional SIM hardware provides a complete solution for the measuring photon count from a sectioned depth layer. From the same SIM raw images, QSIM can provide additional quantitative information about the sample, not available from current commercially-available SIM image processing software. QSIM is expected to facilitate the conversion of SIM from being a qualitative imaging technique into a confocal-like, quantitative imaging modality, and to make it accessible to a broad biomedical research community.

## Availability and requirements

**Project name:** Quantitative structured illumination microscopy.

**Project home page:** http://code.google.com/p/quantitative-sim/.

**Operating systems:** Windows 7 or 8, Mac OS X, and Linux.

**Programming language:** Java.

**Compatible ImageJ versions:** 1.49m or newer.

**Licence:** QSIM is distributed under the terms of the GNU General Public License 2.0.

The QSIM java code is freely available at http://code.google.com/p/quantitative-sim/. Users need to compile this java code in ImageJ to generate a Java class file as instructed in the companion Additional file 1.

## Electronic supplementary material

Additional file 1: **Help file for QSIM.** (PDF 161 KB)
