# Supplementary material for: QSIM: quantitative structured illumination microscopy image processing in ImageJ
Source: Biomed Eng Online. 2015 Jan 14;14:4. doi: 10.1186/1475-925X-14-4 (PMC4360942; doi:10.1186/1475-925X-14-4)
Supplement: Supplementary file 1 — Additional file 1: Help file for QSIM. (PDF 161 KB) [file 12938_2014_934_MOESM1_ESM.pdf]

Help File for QSIM

a. Recommended environments for software operation:
   - Computer operating systems: Windows 7or 8, Mac OS X, and Linux
   - ImageJ versions: 1.49m or newer

b. Because of Java and ImageJ version differences, in order to run QSIM, users need to compile “QSIM_.java” in ImageJ to generate a local “QSIM_.class” file as instructed below:

   (1) Drop “QSIM_.java” file in the “plugins” folder of ImageJ.

   (2) Open “QSIM_.java” in imageJ.

   (3) In the newly opened “QSIM_.java” window, choose File → Compile and Run. This will generate a “QSIM_.class” file in the same folder.

   (4) Restart ImageJ. Choose Plugins → QSIM.
